# Gait Analysis Study Comparing Unicompartmental vs. Total Knee Arthroplasty: Differences in Knee Kinematics

**DOI:** 10.3390/medicina62040648

**Published:** 2026-03-28

**Authors:** Vittorio Castoldi, Andrea Giordano Salvi, Giuseppe Petralia, Giuseppe Aloisi, Pieralberto Valpiana, Alessandro Aprato, Alessandro Massè, Pier Francesco Indelli, Salvatore Risitano

**Affiliations:** 1 Department of Orthopedics and Traumatology, University of Turin, 10126 Turin, Italy; vittorio.castoldi@edu.unito.it (V.C.); ale_aprato@hotmail.com (A.A.); alessandro.masse@unito.it (A.M.); 2 Department of Orthpaedic Surgery, Sudtiroler Sanitatsbertrieb, 39042 Brixen, Italy; andreasalvi88@yahoo.it (A.G.S.); valpianapieralberto@gmail.com (P.V.); pindelli@stanford.edu (P.F.I.); 3 Dipartimento di Medicina Clinica, Sanità Pubblica, Scienze della Vita e dell’Ambiente, Università degli Studi dell’Aquila, 67100 L’Aquila, Italy; giuseppe.petralia@graduate.univaq.it (G.P.); galoisidr@gmail.com (G.A.); 4 Institute of Biomechanics, Paracelsus Medical University (PMU), 5020 Salzburg, Austria; 5 Department of Orthopedic Surgery, Stanford University School of Medicine, Redwood City, CA 94063, USA

**Keywords:** total knee arthroplasty, unicompartmental knee arthroplasty, gait analysis, kinematics, robotics, knee osteoarthritis

## Abstract

Gait analysis study comparing unicompartmental vs. total knee arthroplasty, differences in knee kinematics: a retrospective cohort study. *Background and Objectives:* Total knee arthroplasty (TKA) is an effective treatment for advanced knee osteoarthritis, although functional outcomes may remain suboptimal in many patients. Unicompartmental knee arthroplasty (UKA) often provides better functional recovery but shows lower long-term implant survival. Recently, personalized TKA approaches have been developed to improve kinematic restoration and patient satisfaction. This study aimed to compare knee kinematics among patients who underwent personalized TKA, medial UKA, and healthy controls. *Materials and Methods:* This retrospective cohort study included 9 patients treated with robotic-assisted personalized TKA, 9 patients treated with medial UKA, and 9 healthy controls matched for age, sex, and BMI. Inclusion criteria were age 60–80 years, Kellgren–Lawrence grade III–IV, a minimum follow-up of 12 months, deviation from neutral HKA < 15°, healthy contralateral knee, and high postoperative functional scores. Exclusion criteria included valgus knees (HKA > 180°), postoperative complications, and neuromotor disorders. In the TKA group, a Medial Congruent implant was implanted with ROSA robotic assistance using a restricted kinematic alignment (±5° HKA) and asymmetric intercompartmental balancing. In the UKA group, a fixed-bearing medial implant (Physica ZUK) was used. Gait analysis was performed on a markerless instrumented treadmill (WalkerView™; Dalmine, Italy). Differences between groups were analyzed using one-way ANOVA and Tukey’s post-hoc test (*p* < 0.05). *Results:* UKA patients walked with a stiffer knee during stance. Knee range of motion during stance increased from UKA (6.3° ± 7.2°) to TKA (13.6° ± 8.8°, *p* = 0.045) and to controls (16.6° ± 4.5°, *p* = 0.02). During loading response, UKA patients showed lower flexion (10.2° ± 6.1°) than TKA (19.4° ± 7.9°, *p* = 0.049) and controls (19.6° ± 2.8°, *p* = 0.004). Knee flexion during swing was comparable between UKA and TKA. *Conclusions:* UKA patients demonstrated reduced knee flexion during early stance compared with robotic-assisted TKA and healthy controls. The observed differences may reflect multiple factors, including surgical technique, implant design, and patient-related characteristics. Because preoperative functional data were not available, potential selection bias cannot be excluded. These findings should be interpreted cautiously and warrant confirmation in larger prospective studies.

## 1. Introduction

Knee osteoarthritis (OA) is a leading cause of pain, disability, and reduced quality of life among older adults. Total knee arthroplasty (TKA) represents a well-established and effective treatment for end-stage OA, ensuring pain relief and high implant survival. However, despite excellent survivorship, functional outcomes often remain suboptimal, and up to 19% of patients report postoperative sensations of instability, pain, or stiffness [1,2,3,4,5]. In recent years, growing attention has been directed toward personalized alignment strategies and implant designs in TKA, aiming to improve joint functionality, minimize complications, and enhance patient satisfaction.

Unicompartmental knee arthroplasty (UKA) offers a less invasive alternative that preserves both bone stock and native ligament structures, thereby maintaining more physiological joint kinematics and proprioception. UKA is often associated with faster recovery and better early functional results compared with TKA. Nevertheless, this procedure still shows lower long-term implant survival and higher annual revision rates, restricting its indication to carefully selected patients [6,7,8,9,10,11,12]. Consequently, in patients eligible for both procedures, the optimal surgical choice remains debated and often depends on surgeon expertise and clinical judgment.

Most comparative studies between TKA and UKA have traditionally focused on implant survival, surgical time, intraoperative blood loss, range of motion (ROM), and functional scores [11,13,14]. However, functional outcome scores, commonly based on patient-reported outcome measures (PROMs), have shown variable reliability [15]. Although PROMs such as the *Knee Injury and Osteoarthritis Outcome Score* (KOOS) are widely used to assess quality of life (QoL) and activities of daily living (ADL) [16,17], they do not always reflect objective motor performance. For instance, Ehlers et al. [18] reported that 82% of patients were satisfied after surgery, and satisfaction was significantly associated with improvements in pain and function. Interestingly, even among those without objective functional improvement, 59% still expressed satisfaction, suggesting that subjective perceptions and expectations may outweigh biomechanical recovery in determining postoperative satisfaction.

Similarly, Hill et al. [19] found a weak association between PROMs and standard functional tests (SFTs) in OA and TKA patients. Stevens-Lapsley et al. [20] also demonstrated that KOOSs were mainly driven by pain relief rather than actual performance improvement, particularly one month postoperatively. These findings underscore the need to complement subjective measures with objective assessments of physical function, such as spatiotemporal and kinematic gait parameters (e.g., cadence, stance/swing time, step length, joint ROM) [19,21].

Three-dimensional gait analysis represents the most comprehensive and objective tool for quantifying postoperative function after knee arthroplasty [22]. It allows detailed evaluation of compensatory strategies, stiffness, and instability that may persist despite good clinical scores.

In particular, the *loading response phase*, the early stance period during which the quadriceps eccentrically control knee flexion, is often altered in patients adopting a “quadriceps avoidance gait.” This strategy, characterized by reduced knee flexion during early stance, reflects impaired proprioceptive feedback and reduced quadriceps activation, especially when the anterior cruciate ligament (ACL) is sacrificed. Alterations may also be observed during the *swing phase*, when knee flexion may appear reduced or asymmetric.

These biomechanical deviations can compromise functional recovery and promote compensatory joint loading patterns, potentially influencing long-term outcomes. Therefore, understanding gait mechanics after TKA and UKA is crucial for optimizing surgical selection, improving rehabilitation strategies, and enhancing patient function.

The present study compares quantitative gait analysis parameters between patients undergoing medial UKA, personalized TKA, and healthy controls. The primary aim is to clarify the differences in physiological knee kinematics across groups and to identify characteristic compensatory patterns, particularly the quadriceps avoidance gait, in UKA patients. By objectively describing sagittal knee motion during stance, loading response, and swing phases, this research seeks to deepen understanding of how different surgical approaches affect post-arthroplasty locomotion.

## 2. Materials and Methods

This retrospective cohort study was conducted to compare gait kinematics among patients who underwent total knee arthroplasty (TKA), medial unicompartmental knee arthroplasty (UKA), and healthy control subjects. The study included 9 patients treated with TKA, 9 patients treated with medial UKA, and 9 healthy individuals serving as controls. Participants were matched for age, sex, and body mass index (BMI). All surgical procedures were performed at the same institution by the same orthopedic surgeon to minimize inter-operator variability.

Eligible participants met specific inclusion and exclusion criteria. Inclusion criteria were primary medial knee osteoarthritis graded III–IV according to Kellgren–Lawrence, a deviation from neutral hip–knee–ankle (HKA) angle of less than 15°, a minimum postoperative follow-up of 12 months, and an age range between 60 and 80 years. Further requirements included a native and asymptomatic contralateral knee, high postoperative functional scores (Forgotten Joint Score, FJS > 60; range of motion, ROM 0–125°), and the ability to walk independently without assistive devices. Exclusion criteria comprised valgus knee deformities with an HKA angle greater than 180°, postoperative complications requiring reintervention, and neuromotor disorders potentially influencing gait quality.

Specifically: valgus alignment (HKA > 180°) was excluded because valgus knees are associated with different coronal mechanics and muscle activation patterns that may confound sagittal kinematic comparisons; postoperative complications requiring reintervention were excluded to avoid altered gait due to pain, swelling, or impaired rehabilitation; and neuromotor disorders were excluded because they may independently affect spatiotemporal parameters and joint kinematics regardless of arthroplasty type.

Healthy control participants were voluntarily recruited among physical therapists and residents from the rehabilitation center of Bressanone (Italy). They were recruited based on predefined eligibility criteria, including absence of knee symptoms, no history of lower limb surgery, no neuromuscular disorders, and independent ambulation without assistive devices. All participants underwent gait assessment using the same standardized protocol, on the same instrumented treadmill, supervised by the same physiotherapist, under identical environmental and data-processing conditions to ensure methodological consistency and reduce ascertainment bias.

Postoperative gait analyses were conducted between January 2024 and January 2025 at the Rehabilitation Center of Bressanone (Italy), after a minimum follow-up of 12 months from surgery, under standardized laboratory conditions.

In the TKA group, a Medial Congruent implant (Persona MC, Zimmer Biomet, Warsaw, IN, USA) was implanted with robotic assistance (ROSA, Zimmer Biomet, Warsaw, IN, USA). The surgical goal was to restore the pre-arthritic coronal joint line within safe limits, maintaining a joint line inclination and overall HKA deviation within ±5°, and to reproduce a physiological medio-lateral gap asymmetry with a maximum difference of 2 mm in extension and 4 mm in flexion. In the UKA group, a fixed-bearing medial unicompartmental implant (Physica ZUK, LimaCorporate, San Daniele del Friuli, Italy) was implanted using conventional manual instrumentation.

**Figure 1:**
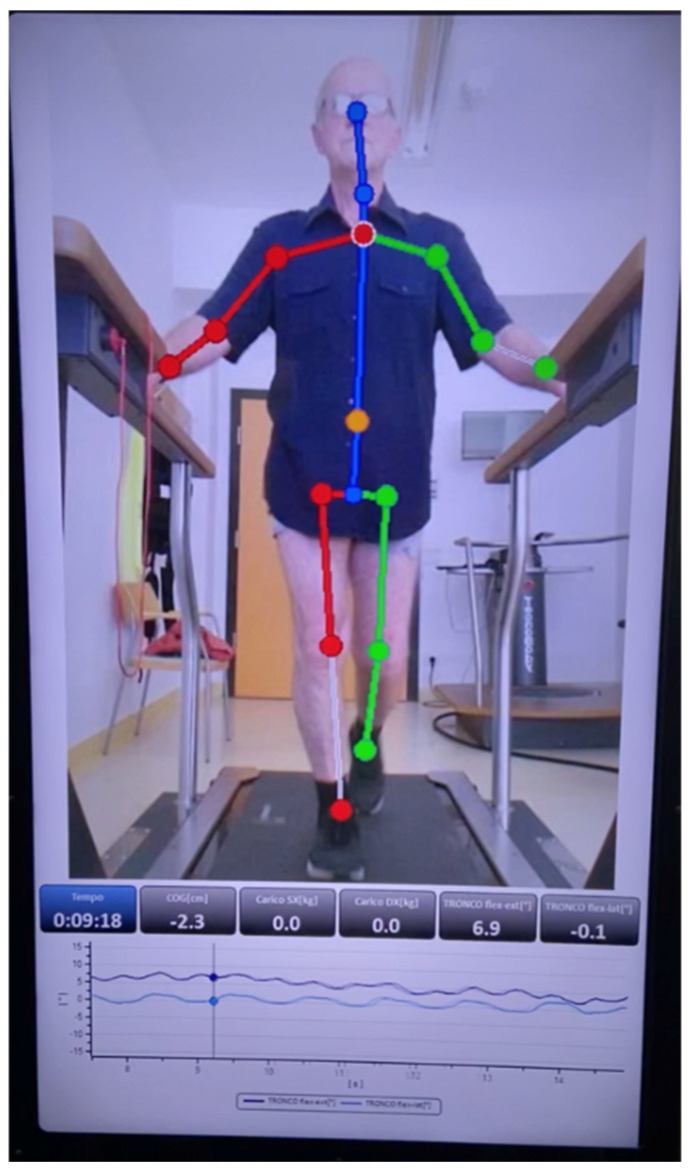
A 69-year-old patient undergoing gait analysis using an instrumented treadmill (WalkerView, TecnoBody MS, Dalmine, Italy). Colored markers represent tracked anatomical landmarks as identified by the system.

Gait analysis was performed by the same physiotherapist using a markerless instrumented treadmill (WalkerView™, TecnoBody S.r.l., Dalmine, Italy), as illustrated in Figure 1. Following a 10-min familiarization session, all participants completed a 3-min walking trial at their self-selected comfortable speed. Data acquisition and test execution were continuously supervised by the same operator to ensure measurement consistency. The WalkerView™ system integrates an optoelectronic sensor and a load-cell platform capable of simultaneously recording kinematic and spatiotemporal parameters. For each participant, mean values were computed over at least 15 consecutive gait cycles to enhance the reliability of the data.

Differences among groups were analyzed using a one-way analysis of variance (ANOVA) to compare mean values and identify statistically significant variations. When a significant main effect was detected, Tukey’s post-hoc test was applied to determine between-group differences while controlling for Type I error. A significance level of *p* < 0.05 was adopted for all analyses. Statistical computations were performed using a standard software package (SPSS, version 26.0; IBM Corp., Armonk, NY, USA).

The study was conducted in accordance with the principles of the Declaration of Helsinki and approved by the institutional ethics committee (IRB:SABES 71/2023). Written informed consent was obtained from all participants prior to data collection.

This study was reported in accordance with the STROBE (Strengthening the Reporting of Observational Studies in Epidemiology) guidelines for cohort studies, and the completed checklist is provided in Supplementary File S1.

## 3. Results

### 3.1. Demographic Data

The demographic characteristics of the three study groups are reported in Table 1. No significant differences were observed in gender distribution or body mass index (BMI) among groups. Healthy controls were significantly younger than both the UKA and TKA groups (*p* < 0.001), while no significant age difference was found between UKA and TKA patients.

### 3.2. Gait Analysis Results

Gait analysis revealed distinct kinematic differences among the three groups. Patients in the UKA group walked with a stiffer knee, showing a lower range of motion throughout the stance phase compared with both TKA patients and healthy controls. The main kinematic parameters recorded during the stance phase of the gait analysis are reported in Table 2.

During the stance phase, the knee range of motion (ROM) progressively increased from the UKA group (6.3° ± 7.2°) to the TKA group (13.6° ± 8.8°, *p* = 0.045) and to healthy controls (16.6° ± 4.5°, *p* = 0.020). Similarly, during the loading response, UKA patients exhibited a significantly lower degree of knee flexion (10.2° ± 6.1°) compared with both TKA patients (19.4° ± 7.9°, *p* = 0.049) and controls (19.6° ± 2.8°, *p* = 0.004). No significant differences were observed among groups for the maximum knee extension during stance. The main kinematic parameters recorded during the swing phase of the gait analysis are reported in Table 3.

During the swing phase, the maximum knee flexion angle was significantly reduced in both arthroplasty groups compared with healthy controls (UKA: 48.1° ± 3.2°; TKA: 45.2° ± 7.1°; controls: 57.2° ± 3.7°; *p* < 0.05), the maximum knee flexion angle was comparable between UKA and TKA groups (48.1° ± 3.2° vs. 45.2° ± 7.1°, *p* = 0.029), indicating similar mobility in this phase of gait.

Overall, UKA patients demonstrated the lowest values of knee flexion and total ROM, while TKA patients showed intermediate values, closer to physiological gait patterns observed in the healthy control group.

Summarizing the study findings, quantitative gait analysis confirmed significant differences in sagittal knee motion among groups, particularly during the early stance and loading response phases. UKA patients exhibited a reduced flexion angle and smaller total ROM during stance, suggesting a stiffer gait pattern compared with both TKA and control subjects. These results provide an objective quantitative basis for subsequent discussion of compensatory gait strategies and functional recovery following different knee arthroplasty procedures.

## 4. Discussion

The present study compared knee kinematics between patients who underwent medial unicompartmental knee arthroplasty (UKA), total knee arthroplasty (TKA), and healthy control subjects using three-dimensional gait analysis. The results demonstrated that UKA patients exhibited a stiffer knee pattern, characterized by reduced flexion during early stance and a smaller range of motion throughout the gait cycle. These findings suggest the presence of a gait pattern resembling quadriceps-avoidance behavior, although the observational design of the study does not allow causal inference regarding the underlying mechanisms.

Quadriceps avoidance gait represents a compensatory adaptation in which the patient minimizes quadriceps activation during the stance phase in order to reduce joint loading. This pattern typically manifests as reduced knee flexion during the loading response and alterations in the normal flexion–extension pattern of the knee during weight acceptance [22,23]. Quantitative gait analysis allows objective identification of such compensatory strategies and may provide clinically relevant insights into postoperative functional recovery.

However, interpretation of these findings should be considered cautiously. Detailed preoperative functional parameters, including preoperative knee range of motion and osteoarthritis severity classification, were not consistently available for all patients. Because UKA and TKA are typically performed according to different clinical indications, the absence of these baseline data may introduce potential selection bias between the surgical cohorts. Consequently, it cannot be definitively determined whether the observed gait differences reflect surgical factors or pre-existing functional differences between patient groups.

Several factors may contribute to the observed differences in postoperative knee kinematics. In particular, surgical technique and implant design may influence postoperative knee kinematics. Previous studies have demonstrated that prosthetic design significantly affects knee motion patterns after total knee arthroplasty [12,13]. Furthermore, robotic-assisted TKA has been proposed to improve implant positioning and soft-tissue balancing, potentially resulting in more physiological gait patterns compared with conventional techniques [22].

These biomechanical factors may partly explain the differences observed between robotic-assisted TKA and manual UKA in the present study.

The preservation of the anterior cruciate ligament in UKA has traditionally been considered advantageous for maintaining more physiological knee kinematics. However, ligament preservation does not necessarily guarantee normal neuromuscular function, particularly in elderly patients in whom age-related neuromotor decline and altered sensorimotor control may influence postoperative gait patterns. These aspects were not directly assessed in the present study and therefore cannot be considered definitive explanatory mechanisms.

Muscle quality may also influence postoperative gait adaptations in elderly patients. However, sarcopenia and neuromuscular function were not directly measured in the present cohort. Therefore, any interpretation regarding their contribution to the observed gait patterns remains speculative. Future investigations incorporating objective muscle assessment techniques, such as ultrasound elastography, could provide quantitative evaluation of quadriceps muscle trophism and stiffness and help clarify their potential role in postoperative gait behavior.

Overall, the findings of this study suggest that gait patterns following knee arthroplasty are likely influenced by multiple interacting factors, including surgical technique, implant design, patient characteristics, and neuromuscular function. Further prospective studies with larger cohorts and detailed preoperative assessments will be required to better clarify the relative contribution of these variables and to determine whether robotic-assisted techniques or implant design features translate into meaningful improvements in functional gait outcomes.

**Take-home messages for clinicians**

- In this selected cohort, UKA patients showed a gait pattern characterized by reduced knee flexion during early stance, whereas robotic-assisted personalized TKA showed gait characteristics closer to those observed in healthy control reference values.
- Objective gait assessment may help identify residual functional deficits even when PROMs and ROM appear satisfactory.
- In older patients, individualized surgical planning and targeted rehabilitation focusing on early-stance knee flexion control may be relevant.

**Take-home messages for researchers**

- Prospective studies with larger cohorts and preoperative baseline gait analysis are needed to confirm whether the observed patterns are attributable to surgical technique versus preexisting gait adaptations.
- Future comparative studies should control for technology (robotic vs. manual) across procedures and include objective muscle strength/sarcopenia measures.
- Validation/measurement-error considerations of markerless systems should be incorporated a priori (e.g., minimal detectable change) when interpreting small kinematic differences.

## 5. Conclusions

This study compared knee kinematics among patients who underwent medial unicompartmental knee arthroplasty (UKA), total knee arthroplasty (TKA), and healthy controls through instrumented gait analysis. The findings revealed that the UKA group exhibited a stiffer gait pattern, characterized by reduced knee flexion during the stance phase and a lower total range of motion, compared with the TKA group and healthy controls. These results suggest that, within the limitations of this exploratory cohort, in elderly individuals, UKA may not fully restore physiological knee kinematics despite its theoretical advantages in ligament preservation.

While personalized and robot-assisted TKA appears to provide a gait pattern closer to normal, further studies are needed to confirm whether these findings translate into better functional outcomes in older patients. Whether these differences reflect surgical technique, patient selection, neuromuscular factors, or measurement variability cannot be definitively determined.

Future research should include larger, prospective cohorts and incorporate pre-operative gait analysis to better tailor both surgical alignment strategies and rehabilitation programs. Integrating robotic-assisted surgery with prehabilitation and targeted nutritional optimization may enhance functional recovery, particularly in sarcopenic and frail elderly populations.

## 6. Limitations

This study presents several limitations that should be acknowledged. First, the retrospective design and the relatively small sample size limit the generalizability of the findings and increase the risk of selection bias, despite matching for age, sex, and BMI. Second, detailed preoperative functional parameters, including knee range of motion and radiographic osteoarthritis severity classification, were not consistently available for all patients. Since UKA and TKA are typically performed according to different clinical indications, the absence of these data may introduce potential selection bias between the surgical cohorts and limit causal interpretation of the observed postoperative gait differences. Third, preoperative gait analysis and detailed baseline kinematic data were not available, preventing direct comparison of pre- and postoperative changes and limiting the ability to determine whether observed differences were preexisting or surgery-related. Fourth, a potential technological confounder exists, as robotic-assisted personalized TKA was compared with manually performed UKA. The absence of robotic assistance in the UKA group may have introduced variability related to implant positioning and soft-tissue balancing. At our institution, robotic assistance is currently implemented for TKA but not routinely for UKA, which explains the technological asymmetry between groups. However, all procedures were performed by the same experienced orthopedic surgeon using standardized surgical protocols, which may have reduced inter-surgeon variability and partially mitigated this potential source of bias. Future research should aim to include robotic-assisted UKA cohorts to directly compare robotic versus manual techniques across both procedures and better isolate the effect of surgical technology from implant design. Fifth, gait assessment was performed using a markerless depth-sensing system (WalkerView™), which, although clinically practical and validated for functional evaluation, does not provide the same level of precision as laboratory-based marker systems. Therefore, small between-group differences should be interpreted cautiously. Finally, sarcopenia, muscle strength, and intrinsic muscle mechanical properties were not directly measured. Therefore, any interpretation regarding neuromuscular mechanisms remains speculative. Future prospective studies with larger cohorts, preoperative assessments, robotic-assisted comparison groups, and objective muscle evaluation—including techniques such as ultrasound elastography to quantify muscle stiffness and trophism—are needed to confirm and expand upon these findings.

## Figures and Tables

**Table 1:** Demographic characteristics of the study population.

	UKA	TKA	Control	Control vs. UKA	Control vs. TKA	UKA vs. TKA
Age (years)	66 ± 6	68 ± 5	36 ± 13	*p* < 0.001	*p* < 0.001	n.s.
Gender (F/M)	4/9	3/9	4/9	n.s.	n.s.	n.s.
BMI (kg/m^2^)	26 ± 2.6	27 ± 3.8	25 ± 3.3	n.s.	n.s.	n.s.

Abbreviations: UKA, unicompartmental knee arthroplasty; TKA, total knee arthroplasty; BMI, body mass index; n.s., not significant.

**Table 2:** Main kinematic parameters recorded during the stance phase of the gait analysis.

	UKA	TKA	Control	Control vs. UKA	Control vs. TKA	UKA vs. TKA
Knee ROM (°)	6.3 ± 7.2	13.6 ± 8.8	16.6 ± 4.5	*p* < 0.05	n.s.	*p* < 0.05
Loading Response (°)	10.2 ± 6.1	19.4 ± 7.9	19.6 ± 2.8	*p* < 0.05	n.s.	*p* < 0.05
Max Knee Extension (°)	4.7 ± 4.0	5.3 ± 4.6	3.0 ± 3.4	n.s.	n.s.	n.s.

Abbreviations: UKA, unicompartmental knee arthroplasty; TKA, total knee arthroplasty; n.s., not significant.

**Table 3:** Main kinematic parameters recorded during the swing phase of the gait analysis.

	UKA	TKA	Control	Control vs. UKA	Control vs. TKA	UKA vs. TKA
Max Knee Flexion (°)	48.1 ± 3.2	45.2 ± 7.1	57.2 ± 3.7	*p* < 0.05	*p* < 0.05	n.s.

Abbreviations: UKA, unicompartmental knee arthroplasty; TKA, total knee arthroplasty; n.s., not significant.

## Data Availability

Data is unavailable due to privacy of patients.

## Supplementary Materials

The following supporting information can be downloaded at: https://www.mdpi.com/article/10.3390/medicina62040648/s1, Figure, legends and tables are attached in the manuscript. Supplementary File S1: Completed STROBE checklist for cohort studies.

## Abbreviations

The following abbreviations are used in this manuscript:

ACL	Anterior Cruciate Ligament
ADL	Activities of Daily Living
ANOVA	Analysis of Variance
BMI	Body Mass Index
FJS	Forgotten Joint Score
fMRI	Functional Magnetic Resonance Imaging
HKA	Hip–Knee–Ankle (angle)
KOA	Knee Osteoarthritis
KOOS	Knee Injury and Osteoarthritis Outcome Score
OA	Osteoarthritis
PROMs	Patient-Reported Outcome Measures
QoL	Quality of Life
ROM	Range of Motion
SPSS	Statistical Package for the Social Sciences
TKA	Total Knee Arthroplasty
UKA	Unicompartmental Knee Arthroplasty
